# Pet cancer cases and patterns of treatment at a Spanish veterinary teaching hospital: a retrospective study from 2015 to 2024

**DOI:** 10.3389/fvets.2025.1588840

**Published:** 2025-07-08

**Authors:** Beatriz Romero, Julen Susperregui, Raquel Díez, E. Milena Vazquez, Cristina Lopez, Raúl de la Puente, M. Jose Diez, Nelida Fernandez, Jose M. Rodriguez-Altonaga, Ana M. Sahagun

**Affiliations:** ^1^Department of Biomedical Sciences, Veterinary Faculty, Institute of Biomedicine (IBIOMED), University of Leon, Leon, Spain; ^2^Applied Mathematics, Department of Mathematics, University of Leon, Leon, Spain; ^3^Department of Veterinary Medicine, Surgery and Anatomy. Director of the Veterinary Teaching Hospital (HVULE), Veterinary Faculty, University of Leon, Leon, Spain

**Keywords:** antineoplastic agent, cancer, chemotherapy, pet, prescription, surgery, veterinary teaching hospital

## Abstract

Cancer is one of the most common causes of death for companion animals. The study aimed to describe the characteristics of the clinical cases of pets attending at the Veterinary Teaching Hospital (University of Leon, Spain) and diagnosed with tumors. A retrospective study was carried out between 2015 and 2024. A total of 123 animals comprising 107 dogs and 16 cats were obtained from the clinical records. A mean annual incidence risk of 530 of 100,000 animals was calculated. Most animals were dogs (87.0%), females (62.6%), purebred (77.2%) and aged (78.9%). Tumors were mainly malignant (87.8%), they were of epithelial origin (40.7%), and mostly located in mammary glands (27.6%) or skin/mucosa (26.8%). Carcinoma (35.8%) and lymphoma (19.5%) were the major histological types. Almost half of the animals underwent surgical treatment (42.3%). Chemotherapy was administered to 37.4% of the animals, mostly by the oral route. QL01E (protein kinase inhibitors) was the main pharmacological group employed. Concomitant treatments and dietary supplements were also used. Euthanasia was applied to 26.8% of the animals.

## 1 Introduction

Cancer is a leading cause of morbidity and death in pets, as a consequence of the recent extension of their lifespan (1–5). According to the Veterinary Cancer Society, one out of four dogs will be diagnosed with cancer in their lifetime (6). Prevention, early detection and treatment of diseases in companion animals, mainly dogs and cats, have contributed to increasing their life expectancy beyond the age established by nature. Medical and social advances and nutrition improvements achieved in recent decades, from which animals also benefit, are also factors that may help to explain this increase (7). Moreover, the unique relationship established between owners and pets, which are often considered “a member of the family” (8, 9), has made owners more inclined to take care for these animals and spend more on them and on those treatments they may need for various diseases (9), cancer among them.

Although this disease is not reportable in these animals, several studies have determined cancer incidence in pets (10–12), and different sources have been used to obtain case records. The incidence rate for malignant cancers in pets ranges from 142.8 (10) to 852/100,000 dogs (11), and from 63 (10) to 412/100,000 cats (12). It should be noted that the recording of tumors in dogs and cats are not completely standardized, and different tools such as VetCompass (13), networks like VetOncoNet (14), national or regional tumor registries (15, 16) or data from referral hospitals (17, 18) have been used to calculate cancer incidence. In other cases the incidence of a particular type of tumor, such as lymphoma (13, 18), cutaneous (19) or mammary gland tumors (17, 20), or salivary neoplasia (21) has been documented.

Regarding treatment possibilities, although guidelines have been established in companion animals (22), there is no information available on their use in field conditions. As sick animals are brought by people of the surrounding areas, Veterinary Teaching Hospitals may be a reliable source of information of animal diseases. They are also referrals for primary care professionals, although information on clinical cases is often used only for accounting purposes, to demonstrate that they have reached a minimum number of clinical cases to be seen by the students regarding the different animal species or clinical services offered. In Great Britain or Australia, databases such as UK VetCompass and Australia VetCompass are available, indexing clinical records around these countries as a way to improve knowledge on identification, prevention, and treatment of diseases in pets (23, 24), sharing this information among practitioners. In other countries little evidence-based data are available to veterinarians to improve animal health and welfare, and only some prescription surveys have been conducted (25, 26). However, it is necessary to have practical and real information about the diseases diagnosed in pets and those treatments carried out on a daily basis. In addition, in the case of cancer, although some veterinary drugs have recently been approved, most of the medicines used are still of human use. Therefore, in this study we have retrospectively evaluated a population of pets diagnosed with cancer in a Spanish Veterinary Teaching Hospital over a 10-year period. The objective was to characterize the population affected, the types of tumor diagnosed, and the modalities of treatment followed.

## 2 Materials and methods

A retrospective review of medical records from the Veterinary Teaching Hospital (HVULE) (Faculty of Veterinary Medicine, University of Leon, Spain), from January 2015 to December 2024, was performed. The Veterinary Teaching Hospital works as a referral hospital for other practitioners in the surrounding area, and carries out diagnostic, clinical and preventive practices. Clinical services in the HVULE are organized according to two main groups of target species: small and large animals, both equipped with emergency facilities. All the teaching staff were qualified veterinarians. The HVULE offers specialty services for oncology diagnosis and treatment such as Diagnostic Imaging, General Surgery, Clinical Pathology or Oncology, among others. The hospital also has a veterinarian responsible for Oncology and Internal Medicine, with part-time collaboration of an external practitioner accredited in Oncology by AVEPA (Spanish Small Animals Veterinary Association). Regarding Surgery in companion animals, three trained veterinarians performed surgeries at the HVULE, and they were accredited by AVEPA in Soft Tissue Surgery, and also recognized at European level. In this study, animals attending at the Small Animals Service were evaluated.

Medical records stored in the veterinary hospital database (GestorVet, Las Palmas de Gran Canaria, Spain) were reviewed and searched for pet patients with a diagnosis of cancer. Potential cases of cancer from the electronic database were identified by searching for different Spanish search terms: cancer, tumor, carcin*, adenocarc*, mastocitoma, sarcoma, *oma, tocera*, carbopl*, ciclof*, cloram*, doxorub*, lomust*, mitoxant*, vincr*, vinbl*, CHOP, COP, *LOP, and UW within 1st January 2015 to 31st December 2024. The retrieved clinical records of these potential cases were then assessed by a veterinarian, and confirmed after having checked diagnosis and treatment. Patients whose medical record lacked essential information (presumptive diagnosis, or absence of diagnosis or treatment) were excluded.

Information collected from medical records included data on signalment, such as age at diagnosis, purebred or not (if purebred, which breed), sex, neuter status, and weight, as well as those characteristics related to tumor diagnosis (malignancy, type of tumor, histological origin of cells, and location). For these latter characteristics data recorded was based on the results of the cytology and/or biopsy, and the advice of a pathologist who usually diagnoses tumors in the HVULE was required. Regarding histogenesis classification, the type of the tumor cells were grouped as epithelial, mesenchymal, or round cells (27). Tumors were coded according to the Vet-ICD-O-canine system (Veterinary International Classification of Diseases for Oncology canine neoplasms, first edition) (28). In this case and to simplify data presentation, tumors were grouped into 7 anatomical sites or locations and 6 histotypes.

Treatment information encompassed those modalities of treatment followed (surgery or chemotherapy) or if the animal was euthanized. Supportive medical treatments were also collected. As for chemotherapy treatment, information included drugs, dosages, administration route, and outcome of treatment (death, cure, disease progression, disease recurrence, unknown). Adverse events were also recorded according to the Veterinary Cooperative Oncology Group-Common Terminology Criteria for Adverse Events (VCOG-CTCAE v2) (29). Personal data such as pet names or information on the owners was not accessed. The age of cases was calculated from their date of birth, and the date at which the first tumor diagnosis was also obtained. Dogs were described as young (< 2 years), mature (2–6 years), senior (7–11 years) and geriatric (> 12 years) (30), whereas cats were grouped as kitten (< 7 months), junior (7 months to 2 years), prime (3–6 years), mature (7–10 years), senior (11–14 years) and geriatric (> 15 years) (31). Weight and breed were used to define the size of the animals. For adult dogs, they were classified as toy and small (< 10 kg), medium (10 to < 20 kg), large (20 to > 40 kg), and giant (≥ 40 kg). Cats were grouped as small (< 2.5 kg) or medium (2.5–6 kg).

The Strengthening the Reporting of Observational Studies in Epidemiology-Veterinary Extension (STROBE-Vet) Statement was used to report data (32).

### 2.1 Statistical analysis

Descriptive statistics (mean, standard deviation, ranges, and frequencies with 95% confidence intervals) were used to characterize this specific pet population. Annual incidence risk with 95% confidence intervals (CI) was estimated by calculating the proportion of incident cases within the total number of dogs and cats under veterinary care at the HVULE from 2015 to 2024 (*n* = 22,987 animals), and the same was made with the animal attendances. The odds ratio (OR) was calculated with their respective 95% CI. Multivariate forward-step logistic regression analysis was also performed to identify those variables potentially associated with the use of surgery as treatment. Model calibration was assessed using the Hosmer-Lemeshow goodness-of-fit test. The statistical package IBM SPSS Statistics 26 (IBM Corporation, Armonk, NY, United States) was used to perform data analysis. A *p* value of 0.05 was always considered as significant.

## 3 Results

The study population consisted of 22,987 companion animals (17,307 dogs and 5,680 cats) under care at the HVULE between 2015 and 2024, attending a total of 63,608 times at the hospital over this period of time. A total of 123 animals were diagnosed with cancer, treated and cared at the HVULE in the 10-year period studied. Mean annual incidence risk was estimated as 530 of 100,000 animals attending at the HVULE over the 10 years studied (range 437–625 of 100,000 animals), 618 of 100,000 dogs (range 501–735 of 100,000 animals), and 264 of 100,000 cats (range 131–398 of 100,000 animals). The annual incidences are also shown in Supplementary Table S1.

Table 1 describes the demographic characteristics of the animals attending at the hospital. Dogs (107 animals) were almost 7 times more represented in the sample than cats (16 patients), and females encompassed 62.6% of the overall cases. In dogs, females (65.4%) almost double the number of males, whereas cats were more homogeneously distributed between males and females. Mean weight at diagnosis was 24.0 ± 13.2 kg in dogs (range 2.9–65.5 kg) and 3.9 ± 1.1 kg in cats (range = 1.5–5.8 kg). Cats tended to be slightly older (9.9 ± 3.8 years) than dogs (9.5 ± 2.7 years), ranging the age of the animals from 2 to 15 years old for dogs, and from 3 to 17 years old for cats. A higher frequency of tumors was seen for senior and geriatric animals, mounting up 78.9% of all tumor cases.

**Table 1:** Demographic characteristics of the animals attending to the HVULE (Spain) and diagnosed with cancer from 2015 to 2024.

	All animals frequency (%) *n* = 123	95% CI	Dogs frequency (%) *n* = 107	95% CI	Cats frequency (%) *n* = 16	95% CI
Animal species						
Dogs	107 (87.0)	0.8105–0.9294	–		–	
Cats	16 (13.0)	0.0706–0.1895	–		–	
Sex						
Female	77 (62.6)	0.5405–0.7115	70 (65.4)	0.5641–0.7443	7 (43.8)	0.1975–0.7012
Male	46 (37.4)	0.2885–0.4595	37 (34.6)	0.2557–0.4359	9 (56.3)	0.2988–0.8025
Neutered status						
Yes	40 (32.5)	0.2424–0.4080	38 (35.5)	0.2645–0.4458	2 (12.5)	0.0155–0.3835
No	83 (67.5)	0.5920–0.7576	69 (64.5)	0.5542–0.7355	14 (87.5)	0.6165–0.9845
Breed status						
Purebreed	95 (77.2)	0.6983–0.8465	79 (73.8)	0.6550–0.8216	16 (100.0)	
Mixed	28 (22.8)	0.1535–0.3017	28 (26.2)	0.1784–0.3450	–	
Size						
Toy and small	22 (17.9)	0.1111–0.2466	21 (19.6)	0.1210–0.2715	1 (6.3)	0.0016–0.3023
Medium	47 (38.2)	0.2962–0.4680	32 (29.9)	0.2123–0.3858	15 (93.8)	0.6977–0.9984
Large	47 (38.2)	0.2962–0.4680	47 (43.9)	0.3452–0.5333	–	
Giant	7 (5.7)	0.0160–0.9709	7 (6.5)	0.0186–0.1126	–	
Age						
Young	2 (1.6)	0.0061–0.0386	–		–	
Prime	–		–		2 (12.5)	0.0155–0.3835
Mature	24 (19.5)	0.1251–0.2652	13 (12.1)	0.0596–0.1834	11 (68.8)	0.4134–0.8898
Senior	67 (54.5)	0.4567–0.6327	64 (59.8)	0.5052–0.6910	3 (18.8)	0.0405–0.4565
Geriatric	30 (24.4)	0.1680–0.3198	30 (28.0)	0.1953–0.3655	–	

CI, confidence interval.

The majority of the animals were medium to large in size. Dogs were mostly large-giant (43.9%) or medium-sized animals (38.2%), whereas practically all cats were of medium size (93.8%). Purebred animals were in the majority in both species, accounting for 3 quarters of dogs (73.8%) and all cats. With regard to animal breeds, there was a greater variety of breeds among dogs, with a number of animals ranging in many of them from 1 to 6 individuals. Tumors were most frequently diagnosed in Golden Retriever and Boxer breeds (6.5% each) followed by German shepherds and Labrador Retriever (5.6%). As for cats, although with a much smaller number of animals, more than three quarters of them belonged to the European shorthair cat, and the other 3 animals were each of a different breed (Maine Coon, Bombay and Siamese).

Table 2 summarizes the characteristics of the tumors diagnosed in these animals. Moreover, the classification according to the Vet-ICD-O-canine system is presented in Supplementary Table S2. Benign tumors were found in only 12.2% of patients. Mean age was nearly similar in those dogs diagnosed for benign (9.8 ± 2.5 years old) and malignant tumors (9.5 ± 2.7 years). Something similar occurred in cats, as the only animal with a benign tumor was 10 years old, and the age in feline animals with malignant tumors was 10.0 ± 3.8 years old. Epithelium was the predominant tissue of origin (40.7%), followed by those that had their origin in round cells (30.1%). The most frequent histotype was carcinoma (35.8%), followed by lymphoma (19.5%), and mast cell tumors (10.6%). In this characteristic, the group “other” accounts for 22.0%, and includes different typologies with lower frequencies. Of the carcinomas, 59.1% were present in the mammary gland. As for topography, the three most affected systems were mammary glands (27.6%), skin/mucosa (26.8%), and the hematolymphoid system (17.9%), although proportions changed from one species to another. All the animals with mammary tumors were female. Regarding carcinoma, female dogs were 4 times more likely to developed this last histotype than males (OR = 3.936; 95% CI = 1.476–10.501; *p* = 0.006); and it was also 3 times more likely for mixed than purebreed dogs (OR = 2.959; 95% CI = 1.159–7.555; *p* = 0.023). In the case of lymphoma, no differences were found according to sex, breed or age but was higher in purebreed (83.3%), females (61.1%) and senior (44.4%) dogs.

**Table 2:** Characteristics of the tumors diagnosed in pets attending to the HVULE (Spain) from 2015 to 2024.

	All animals frequency (%) *n* = 123	**95% CI**	Dogs frequency (%) *n* = 107	95% CI	Cats frequency (%) *n* = 16	95% CI
Malignancy						
Benign	15 (12.2)	0.0641–0.1798	14 (13.1)	0.0669–0.1947	1 (6.3)	0.0016–0.3023
Malignant	108 (87.8)	0.8202–0.9359	93 (86.9)	0.8053–0.9331	15 (93.8)	0.6977–0.9984
Origin						
Epithelial	50 (40.7)	0.3197–0.4933	45 (42.1)	0.3270–0.5141	5 (31.3)	0.1102–0.5866
Mesenchymal	9 (7.3)	0.0271–0.1192	8 (7.4)	0.0249–0.1246	1 (6.3)	0.0016–0.3023
Round cells	37 (30.1)	0.2198–0.3819	30 (28.0)	0.1953–0.3655	7 (43.8)	0.1975–0.7012
Other	27 (22.0)	0.1464–0.2927	24 (22.4)	0.1453–0.3033	3 (18.8)	0.0405–0.4565
Histological type						
Adenoma	6 (4.9)	0.0107–0.0868	6 (5.6)	0.0125–0.0997	–	
Carcinoma	44 (35.8)	0.2730–0.4424	39 (36.4)	0.2733–0.4557	5 (31.3)	0.1102–0.5866
Lymphoma	24 (19.5)	0.1251–0.2652	18 (16.8)	0.0973–0.2391	6 (37.5)	0.1520–0.6457
Mast cells	13 (10.6)	0.0514–0.1600	12 (11.2)	0.0524–0.1719	1 (6.3)	0.0016–0.3023
Sarcoma	9 (7.3)	0.0271–0.1192	8 (7.5)	0.0249–0.1246	1 (6.3)	0.0016–0.3023
Other	27 (22.0)	0.1464–0.2927	24 (22.4)	0.1453–0.3033	3 (18.8)	0.0405–0.4565
Location						
Bone	8 (6.5)	0.0215–0.1086	7 (6.5)	0.0186–0.1123	1 (6.3)	0.0016–0.3023
Mammary gland	34 (27.6)	0.1974–0.3555	32 (29.9)	0.2123–0.3858	2 (12.5)	0.0155–0.3835
Digestive	6 (4.9)	0.0107–0.0868	6 (5.6)	0.0125–0.0997	-	
Hematolymphoid system	22 (17.9)	0.1111–0.2466	16 (15.0)	0.0820–0.2171	6 (37.5)	0.1520–0.6457
Skin/mucosa	33 (26.8)	0.1900–0.3466	27 (25.2)	0.1700–0.3346	6 (37.5)	0.1520–0.6457
Urinary	4 (3.3)	0.0012–0.0639	4 (3.7)	0.0014–0.0733	-	
Other	16 (13.0)	0.0706–0.1895	15 (14.0)	0.0744–0.2060	1 (6.3)	0.0016–0.3023

CI, confidence interval.

The clinical characteristics of the animals diagnosed with a tumor are shown in Table 3. They attended 318 times (range 1–21 visits) at the HVULE during the 10 years assessed, most of them only once (25.8%). The frequency of diagnosis was not constant throughout the study period, being concentrated between 2017 and 2019 (19.5; 21.1 and 16.3%, respectively). Approximately half of the animals (42.3%) underwent surgical treatment. For those who were hospitalized (40.7%), the length of the stay was 2.9 ± 3.5 days (range 1–24 days; median 2). Two thirds of those hospitalized had undergone surgery (68.0%). Euthanasia was carried out in 26.8% of the animals, and almost half of them (42.4%) did not receive any treatment at all. Table 4 shows the logistic analysis performed to identify those variables associated with surgery as therapeutic treatment in those animals diagnosed with tumors. Logistic regression was performed with all the animals diagnosed and only with dogs. Model calibration was good in both models (all animals and only dogs), as shown by the Hosmer-Leweshow goodness-of-fit test (χ^2^ = 7.503, *p* = 0.277 when all animals were considered; and χ^2^ = 9.470, *p* = 0.149 if only dogs were included). Moreover, the observed:expected ratio was 77% for the model defined with all the animals, and 78.5% when only dogs were considered. When the animals were considered as a whole, the likelihood of following surgical treatment was significantly 4.4 times higher in female animals, 6.4 times higher if a benign tumor was detected, 15.0 times higher if the animal had been hospitalized, and 6.3 times higher if no chemotherapy was used to treat the disorder. Furthermore, a negative correlation was obtained with the year of the first attendance at HVULE, as over the years the number of cases treated with surgery was decreasing. If only dogs were included in the logistic analysis, the probability of being treated with surgery was 3.8 times higher in female animals, 22.2 times higher if animals had been hospitalized, and 11.3 times higher in those animals in which chemotherapy was not employed as treatment modality. Again, a negative correlation was observed with the year of the first attendance to HVULE.

**Table 3:** Clinical characteristics and treatment modalities administered to pets attending to the HVULE (Spain) from 2015 to 2024 and diagnosed with tumors.

	All animals frequency (%) *n* = 123	95% CI	Dogs frequency (%) *n* = 107	95% CI	Cats frequency (%) *n* = 16	95% CI
Surgery						
Yes	52 (42.3)	0.3355–0.5101	47 (43.9)	0.3452–0.5333	5 (31.3)	0.1102–0.5866
No	71 (57.7)	0.4899–0.6645	60 (56.1)	0.4667–0.6548	11 (68.8)	0.4134–0.8898
Chemotherapy						
Yes	46 (37.4)	0.2885–0.4595	41 (38.3)	0.2911–0.4753	5 (31.3)	0.1102–0.5866
No	77 (62.6)	0.5405–0.7115	66 (61.7)	0.5247–0.7089	11 (68.8)	0.4134–0.8898
Hospitalization						
Yes	50 (40.7)	0.3197–0.4933	42 (39.3)	0.3000–0.4850	8 (50.0)	0.2465–0.7535
No	73 (59.3)	0.5067–0.6803	65 (60.7)	0.5150–0.7000	8 (50.0)	0.2465–0.7535
Days of hospitalization (n = 50)						
1–2	32 (64.0)	0.5070–0.7730	32 (76.2)	0.6331–0.8907	-	
3–4	11 (22.0)	0.1216–0.3584	6 (14.2)	0.0370–0.2487	6 (75.0)	0.3491–0.9681
≥ 5	7 (14.0)	0.0299–0.2101	4 (9.5)	0.0065–0.1840	2 (25.0)	0.0319–0.6509
Euthanasia						
Yes	33 (26.8)	0.1900–0.3466	28 (26.2)	0.1784–0.3450	5 (31.3)	0.1102–0.5866
No	90 (73.2)	0.6534–0.8100	79 (73.8)	0.6550–0.8216	11 (68.8)	0.4134–0.8898
Year of first attendance to HVULE						
2015	8 (6.5)	0.0215–0.1086	6 (5.6)	0.0125–0.0997	2 (12.5)	0.0155–0.3835
2016	12 (9.8)	0.0451–0.1500	9 (8.4)	0.0315–0.1367	3 (18.8)	0.0405–0.4565
2017	24 (19.5)	0.1251–0.2652	20 (18.7)	0.1130–0.2608	4 (25.0)	0.0727–0.5238
2018	26 (21.1)	0.1392–0.2835	23 (21.5)	0.1371–0.2928	3 (18.8)	0.0405–0.4565
2019	20 (16.3)	0.0974–0.2278	20 (18.7)	0.1130–0.2608	-	
2020	4 (3.3)	0.0012–0.0639	3 (2.8)	0.0032–0.0593	1 (6.3)	0.0016–0.3023
2021	11 (8.9)	0.0390–0.1399	9 (8.4)	0.0315–0.1367	2 (12.5)	0.0155–0.3835
2022	10 (8.1)	0.0330–0.1296	10 (9.3)	0.0383–0.1486	-	
2023	5 (4.1)	0.0058–0.0756	4 (3.7)	0.0014–0.0733	1 (6.3)	0.0016–0.3023
2024	3 (2.4)	0.0029–0.0517	3 (2.8)	0.0032–0.0593	-	
No. of attendances to HVULE						
1–2	96 (78.0)	0.7073–0.8536	83 (77.6)	0.6967–0.8547	13 (81.3)	0.5435–0.9595
3–6	9 (7.3)	0.0271–0.1192	7 (6.5)	0.0186–0.1123	2 (12.5)	0.0155–0.3835
≥ 7	18 (14.6)	0.0839–0.2088	17 (15.9)	0.0896–0.2281	1 (6.3)	0.0016–0.3023

CI, confidence interval.

**Table 4:** Multivariate logistic regression analysis of demographic and clinical characteristics relevant to the selection of surgery as treatment.

Variable	All animals		Dogs	
	OR (CI 95%)	*p*-value	OR (CI 95%)	*p*-value
Year *	1.002 (1.001–1.003)	< 0.001	1.002 (1.001–1.003)*	< 0.001
Sex (female)	4.367 (1.542–12.366)	0.006	3.828 (1.234–11.879)	0.020
Malignancy (benign)	6.389 (1.359–30.043)	0.019	–	–
Hospitalization (yes)	15.022 (4.982–45.294)	< 0.001	22.153 (5.831–84.153)	< 0.001
Chemotherapy (no)	6.285 (2.042–19.339)	0.001	11.259 (2.909–43.580)	< 0.001

OR, odds ratio; CI, confidence interval. * Year of first attendance at HVULE was negatively correlated with the use of surgery as modality treatment.

Chemotherapy was prescribed to 46 animals (37.4%), with only 5 cats treated with this modality. Both treatments (surgery and chemotherapy) were combined in 10 animals (8.1%). Table 5 listed cytotoxic agents administered to pets, according to ATC and ATCvet classifications (33, 34). A total of 74 different chemotherapeutic treatments were prescribed to animals (median = 1; range 1–19 treatments/animal), and they included 11 different drugs. Single-agent chemotherapy was used in more than two thirds of the animals (69.6%). Most of these treatments were administered orally (53.2%), and the rest by the intravenous route. In dogs, intravenous treatments included mitoxantrone, doxorubicine, vincristine, vinblastine and carboplatin. Among oral medications, the predominant drug was toceranib, but other active ingredients such as cyclophosphamide, lomustine, chlorambucil, melphalan and masitinib were also used. In cats, intravenous treatments were based on carboplatin and vincristine, and oral ones in chlorambucil, cyclophosphamide and toceranib. Moreover, metronomic chemotherapy was administered to 4 animals (8.7%): 3 dogs (2 received toceranib and 1 animal cyclophosphamide) and 1 cat (toceranib). Medicinal products approved for human use were mostly prescribed (72.3%). Regarding chemotherapeutic protocols, they were very varied. In fact, up to 24 different protocols were used. These protocols were administered either to only one (36.9%) or two animals (26.1%). Only toceranib was administered to 17 patients (37.0%), usually in neoplasias other than the approved indication (non-resectable canine mast cell tumors). In most of these animals, this drug was given in alternating days with an NSAID except for 4 animals, which received only toceranib.

**Table 5:** Antineoplastic agents prescribed to pets treated at HVULE (Spain) from 2015 to 2024.

Antineoplastic agents ATC/ATCvet	Frequency (%) *n* = 220
Alkylating agents	56 (25.5)
L01AA01 Cyclophosphamide	25 (11.4)
L01AA02 Chlorambucil	9 (4.1)
L01AA03 Melphalan	18 (8.2)
L01AD02 Lomustine	4 (1.8)
Plant alkaloids and other natural products	54 (24.6)
L01CA01 Vinblastine	16 (7.3)
L01CA02 Vincristine	38 (17.3)
Cytotoxic antibiotics and related substances	41 (18.6)
L01DB01 Doxorubicin	29 (13.2)
L01DB07 Mitoxantrone	12 (5.5)
Protein kinase inhibitors	61 (27.7)
QL01EX06 Masitinib	2 (0.9)
QL01EX90 Toceranib	59 (26.8)
Other antineoplastic agents	8 (3.6)
L01XA02 Carboplatin	8 (3.6)

Regarding concomitant treatments (Table 6), the most commonly used drugs were antiemetics (23.5%), followed by corticosteroids for systemic use (21.9%), antibacterials for systemic use (16.2%), and antiinflammatory and antirheumatic products (13.2%). Unlike in the case of cytotoxic drugs, veterinary medicines were mostly employed (66.3%). Moreover, dietary supplements were recommended to owners during and after chemotherapeutic treatment (41.3%) to protect gastric mucosa (89.5%), liver (36.8%), or as a probiotic (10.5%).

**Table 6:** Concomitant treatments prescribed to pets treated with antineoplastic at HVULE (Spain) from 2015 to 2024.

Concomitant treatments ATC/ATCvet	Frequency (%) *n* = 502
Antiemetics and antinauseants	
QA04AD90 Maropitant	118 (23.5)
Drugs for peptic ulcer and gastro-oesophageal reflux disease (GORD)	
A02BC01 Omeprazole	35 (7.0)
A02BA03 Famotidine	12 (2.4)
Bile therapy	
A05AA02 Ursodeoxycholic acid	1 (0.2)
Vitamins	
A11CC04 Calcitriol	3 (0.6)
A11CC05 Colecalciferol	3 (0.6)
Diuretics	
QC03CA01 Furosemide	23 (4.6)
QC03DA01 Spironolactone	1 (0.2)
Antiseptics and disinfectants	
D08AF01 Nitrofural	1 (0.2)
Corticosteroids for systemic use	
QH02AB04 Methylprednisolone	20 (4.0)
QH02AB07 Prednisolone	24 (4.8)
H02AB07 Prednisone	66 (13.1)
Antibacterials for systemic use	
QJ01AA02 Doxycycline	2 (0.4)
J01CA01 Ampicillin	1 (0.2)
QJ01CA04 Amoxicillin	1 (0.2)
QJ01CR02 Amoxicillin & beta-lactamase inhibitor	13 (2.6)
QJ01DD91 Cefovecin	1 (0.2)
QJ01EW11 Sulfamethoxazole and trimethoprime	2 (0.4)
QJ01MA90 Enrofloxacin	6 (1.2)
QJ01MA93 Marbofloxacin	2 (0.4)
QJ01XD01 Metronidazole	53 (10.6)
Antiinflammatory and antirheumatic products	
M01AC01 Piroxicam	20 (4.0)
QM01AC06 Meloxicam	3 (0.6)
QM01AH90 Firocoxib	22 (4.4)
QM01AH91 Robenacoxib	18 (3.6)
QM01AH93 Cimicoxib	2 (0.4)
QM01AX93 Grapiprant	1 (0.2)
Drugs affecting bone structure and mineralization	
M05BA08 Zoledronic acid	1 (0.2)
Opioids	
N02AB03 Fentanyl	3 (0.6)
QN02AX02 Tramadol	13 (2.6)
Other analgesics and antipyretics	
N02BB02 Metamizole	5 (1.0)
N02BE01 Paracetamol	3 (0.6)
N02BF01 Gabapentin	6 (1.2)
QN02BG91 Bedinvetmab	3 (0.6)
Antiepileptics	
QN03AA02 Phenobarbital	3 (0.6)
Antipsychotics	
QN05AA04 Acepromazine	1 (0.2)
Psychoanaleptics	
QN06AX11 Mirtazapine	1 (0.2)
Antihistamines for systemic use	
R06AA02 Diphenhydramine	9 (1.8)

As for the adverse events caused by antineoplastic agents and classified according to VCOG-CTCAE v2 (29), they were recorded in 20 (1 cat and 19 dogs) of those 46 animals following chemotherapeutic treatment (43.5%), with neutropenia (70%) and vomiting (55%) as the most frequent, followed by colitis (35%), anemia, diarrhoea and lethargy (each one 30%). Grade 5 neutropenia was reported in 2 dogs, both treated with lomustine and euthanized. Of the 20 animals developing adverse events, 55% were treated in polytherapy and the rest in monotherapy (Supplementary Table S3). An overdose of toceranib was also detected in one dog, leading to discontinuation of treatment.

Disease outcome after chemotherapy was grouped into 5 categories. In almost 4 out of 10 animals the outcome of treatment was unknown (18 animals; 2 cats). Mortality was also high, as 13 animals (28.2%; 2 cats) unfortunately died, whereas a progression of the disease was observed in 12 animals (26.1%; 1 cat). Finally, disease recurrence was recorded in 2 cases (4.3%), and only 1 animal appeared to be cured, as no further information on the treated tumor was included in its subsequent medical record. The last 3 animals (recurrence or cure) underwent both surgical and chemotherapeutic treatments.

## 4 Discussion

Up to the knowledge of the authors, this is the first study aimed at investigating the characteristics of tumors diagnosed in companion animals over a long period of time (10 years), with a special focus on the pattern of treatments for this disease. The availability of similar studies in literature is really scarce, and they have carried out partial studies of either the characteristics of tumors in dogs or cats, or the description of the prescribed cytotoxic agents. A University clinical setting was used due to the ease of access to clinical information, but also because it is considered a referral center in the geographical area considered.

Cancer has become a major disease in companion animals and one of the leading causes of pet mortality (10). Studies such ours may contribute to a better knowledge of its epidemiology in small animals and the actual treatment options carried out by practitioners. The low number of cases reported in this paper may be partly attributed to the hospital’s location, in a medium-sized city. Other authors have also obtained a low ratio of clinical cases focused on animals diagnosed with cancer and treated against this disease (26).

Tumors were mostly diagnosed in aged animals, which is consistent with data reported by other authors (28, 35, 36). On the other hand, in our study a clearly much smaller number of tumors has been diagnosed in cats, which could be related to the less often attendance to veterinary hospitals of cat’s owners (28), although in Spain the number of cats registered as pets is slightly more than half the number of dogs (37).

The higher incidence in purebred dogs observed is consistent with the literature (16, 38–40), which would be associated to a genetic predisposition in purebred animals, and heritable risks associated with this disease due to inbreeding (7). As for the size of the animals, other studies have indicated that small dogs live longer than large animals and developed lower cancer rates (41, 42). In our study, tumor frequency in large/giant-sized animals was not higher than in small and medium ones, although this fact may be related to the owner preferences for certain breeds in the geographical area where the HVULE is located.

In agreement with data reported elsewhere (36), malignant neoplasms were the most prevalent in our study, being the predominant tissue of origin the epithelial one. Regarding tumor location, other authors have indicated that the most common affected organs/systems were also the mammary gland and the skin (15, 16, 38, 43, 44), which is consistent with our results. Specifically in female dogs, we have observed a high incidence of mammary tumors, which is in agreement with other researchers (10, 16, 45). Mammary gland tumors are the most common neoplasms in intact female dogs, accounting for over 40% of all tumors (46). As stated by Pinello et al. (14), female dogs are at higher risk of developing tumors than male animals, but the same does not occur with cats. A high proportion of lymphomas has also been evidenced in cats, more prone to this type of tumor (28), although in our study the number of cats is very low, and these data should be interpreted with caution.

Treatment choice was based on case-by-case clinical judgement. Nevertheless, the final decision was always made on a shared basis with the owners. As expected, surgery was the most common modality of cancer treatment implemented at the HVULE. In veterinary medicine, it is considered the most important therapeutic option in pets to improve their quality of life (47). As for chemotherapy, this treatment has been prescribed in slightly more than one third of patients, with a lower proportion than surgery. On the other hand, a quarter of the animals were euthanized. Of those, euthanasia was recommended for 14 animals, which did not receive any treatment; surgery was previously carried out in 9; chemotherapy was administered to 10 animals, and 2 received both surgical and cytotoxic treatments before euthanasia was applied.

Regarding chemotherapy, antineoplastic agents have been used to treat pet neoplasias for more than 60 years. Although the use of chemotherapy is more and more common in veterinary medicine, it is not without debate, as it is a form of palliative care to prolong the animal life, and it may have potential severe side effects (3). Cave et al. pointed out that the use of cytotoxic drugs was infrequent among British veterinarians, with a median frequency of at least once every 3 months (48), which would be consistent with our results.

Unlike in our study, other authors reported a higher use of intravenous cytotoxic treatments in pets in comparison with oral treatments (26, 48). It should be noted that in recent years more oral antitumor veterinary drugs have become available (toceranib, and masitinib). In the case of toceranib, although this tyrosine kinase inhibitor is licensed for mast cell tumors, it has also been used by veterinary oncologists for multiple neoplastic diseases (49), as occurred in our study, which would explain its increasing importance in pet treatments. Intravenous medications were always used off-label. Regarding the molecules employed, in a survey carried out in the UK, the most widely prescribed antineoplastic agents were cyclophosphamide and vincristine (48), which may reflect their use against lymphomas, according to the opinion of these authors. Tanaka et al. (26) observed that carboplatin, vincristine and doxorubicine were the most administered drugs in two Japanese Veterinary Teaching Hospitals, pointing out that both doxorubicine and vincristine were also indicated for the treatment of lymphomas. These results are consistent with ours.

Respecting concomitant medications, the administration of antiemetics is common in these treatments, and has allowed to improve the quality of life of pet patients, as well as to better withstand the effects of antineoplastic therapy. Substance P (Neurokinin-1 receptor) antagonist maropitant has become the antiemetic of choice in veterinary patients for the prevention of chemotherapy-induced vomiting (50). NSAIDs and glucocorticoids are common adjuvant treatments for different cancers in veterinary medicine as well. Glucocorticoids are part of several protocols (CHOP, CLOP, COP and LOP), and sometimes are also used as a chemotherapy single agent, due to their ability to inhibit DNA synthesis (22). As for NSAIDs, apart from their usual indications, they are also used when metronomic chemotherapy is performed, and due to their ability to inhibit cyclooxygenase isoform-2 (COX-2), whose expression is considered a negative prognostic factor in various types of canine and feline tumors (51). This inhibitory effect compromises endothelial cell tube formation and VEGF expression, preventing tumor progression (52). Regarding antibacterials, metronidazole was the most prescribed, specifically against diarrhea (53). However, for other authors the used of metronidazole as first-line treatment would not be justified in dogs with chemotherapy-induced diarrhea (54), as this drug significantly reduces in dogs bacterial diversity indices, alters the microbiome composition, and may increase the risk of occurrence of nosocomial or opportunistic infections with microbial resistance (55). Metronidazole and chemotherapy affect *Clostridium* cluster *IV* and *XIVa*, which are known to positively affect the gut health through improved nutrient absorption, production of short chain fatty acids with antiinflammatory properties and epithelial maturation (55). These treatments were also consistent with the adverse reactions observed in the animals. Veterinary medicines were mostly employed as concomitant treatments, which is in agreement with European regulations (56).

Regarding disease outcome after chemotherapeutic treatment, almost a third of the animals died (13 cases). These animals had a wide variety of tumors, and only 2 were underwent surgery. So, chemotherapy treatment in these cases should be considered as palliative, which would improve the quality and length of life of the patient, but ultimately most animals will relapse and die. On the other hand, for a significant proportion of the animals (39.1%) the information was missing. It should be taken into account in these latter cases that these animals may have been followed up in another veterinary clinic, which makes it impossible to know the final result of the treatment, or the animals may have had a fatal outcome, not reported by the owners.

The study is prone to have limitations associated with its retrospective nature. First, some medical records were excluded because of missing information. Second, data were obtained from a veterinary hospital located in a defined geographical area, and may limit the possibility of providing a broader picture of the actual situation regarding the characteristics and treatment of cancer in pets. In this sense, it should be noted that the number of feline cases was very small, which may affect the statistical results in this species and, therefore, the generalizability of our findings. Additionally, the worst moment of COVID-19 pandemic occurred in 2020, and may have had an impact on hospital visits. On the other hand, clinical decisions may be influenced by various factors such as the severity of the disease or the age of the animals. The socioeconomic status of the owners should be also considered, due to the cost of diagnostic and therapeutic procedures. However, and despite these limitations, the current study provides information not previously evaluated in veterinary medicine, and may be a background for further studies.

## 5 Conclusion

This is the first study that describes the pattern of diagnosis and treatment of cancer in pets at a Veterinary Teaching Hospital. We have observed that most tumors have been described in dogs, aged, purebred and female animals. Tumors were usually malignant, and were mostly located in the mammary glands or the skin, being the main histological type carcinoma. The study also provides an actual insight into the treatment modalities mainly followed in pet patients, with surgery as the major therapeutic option employed. Regarding cytotoxic drugs, just over half of the treatments were administered orally (mainly toceranib), whereas intravenous treatments were used off-label. Finally, slightly more than a quarter of the animals were euthanized.

## Data Availability

The raw data supporting the conclusions of this article will be made available by the authors, without undue reservation.
