# Supplementary material for: Pet cancer cases and patterns of treatment at a Spanish veterinary teaching hospital: a retrospective study from 2015 to 2024
Source: Front Vet Sci. 2025 Jul 8;12:1588840. doi: 10.3389/fvets.2025.1588840 (PMC12279513; doi:10.3389/fvets.2025.1588840)
Supplement: Supplementary file 1 [file Data_Sheet_1.docx]

Supplementary table S1. Annual incidence of cancer cases for dogs, cats and all animals in the HVULE.

|  | **Dogs** (100,000 animals) | | **Cats** (100,000 animals) | | **All** (100,000 animals) | |
| --- | --- | --- | --- | --- | --- | --- |
| **Year** | **Incidence** | **95% CI** | **Incidence** | **95% CI** | **Incidence** | **95% CI** |
| 2015 | 240.3 | 48-432 | 200.6 | 0-478 | 229 | 70-387 |
| 2016 | 389.1 | 135-643 | 331.1 | 0-705 | 372.8 | 162-583 |
| 2017 | 942.5 | 531-1354 | 514.8 | 12-1018 | 827.9 | 498-1158 |
| 2018 | 1202.9 | 714-1692 | 296.7 | 0-707 | 966.7 | 590-1344 |
| 2019 | 1420.5 | 802-2039 | - | - | 1040 | 587-1493 |
| 2020 | 183.8 | 0-392 | 168.6 | 0-499 | 179.8 | 4-356 |
| 2021 | 559.7 | 195-924 | 483.1 | 0-1151 | 544 | 223-865 |
| 2022 | 654.5 | 250-1059 | - | - | 526.9 | 201-853 |
| 2023 | 296.3 | 6-586 | 450.5 | 0-1331 | 318.1 | 40-596 |
| 2024 | 320.2 | 0-682 | - | - | 261.1 | 0-556 |
| Total | 618.2 | 501-735 | 264.1 | 131-398 | 530.7 | 437-625 |

CI: confidence interval.

Supplementary table S2. Vet-ICD-O canine classification of tumor type of cancer pets in the HVULE

Supplementary table S3. Adverse events related to antineoplastic according to VCOG-CTACAE criteria (29).

| **Dog** | **Treatment/Protocol** | **Adverse events and grade** |
| --- | --- | --- |
| 1 | Doxorubicin | Neutropenia grade 1 Lymphocytosis grade 3 Vomiting grade 1 |
| 2 | Doxorubicin | Neutropenia grade 1 Haemoglobin grade 2 Thrombocytopenia grade 2 Diarrhoea grade 1 |
| 3 | Doxorubicin | Neutropenia grade 2 Vomiting grade 1 |
| 4 | Mitoxantrone | Neutropenia grade 1 Haemoglobin grade 1 Thrombocytopenia grade 1 Vomiting grade 1 Colitis grade 3 Lethargy/fatigue grade 2 |
| 5 | Mitoxantrone | Haemoglobin grade 2 Thrombocytopenia grade 3 Vomiting grade 2 Colitis grade 3 Lethargy/fatigue grade 2 |
| 6 | UW 25 | Neutropenia grade 2 Haemoglobin grade 1 Thrombocytopenia grade 1 Vomiting grade 1 Enteritis grade 1 |
| 7 | Cyclophosphamide | Neutropenia grade 1 Diarrhoea grade 1 Gastric ulceration grade 2 Lethargy/fatigue grade 1 |
| 8 | Vinblastine Carboplatin Mitoxantrone Chlorambucil | Vomiting grade 1 Lethargy/fatigue grade 1 |
| 9 | CHOP LOP CLOP | Neutropenia grade 5 Vomiting grade 1 |
| 10 | COP CHOP | Neutropenia grade 3 |
| 11 | CHOP Lomustine | Neutropenia grade 3 |
| 12 | Doxorubicin Cyclophosphamide Toceranib | Vomiting grade 1 Polyuria grade 1 Incontinence, urinary grade 2 |
| 13 | Lomustine Toceranib | Lethargy/fatigue grade 1 |
| 14 | Vinblastine Toceranib | Vomiting grade 1 Diarrhoea grade 2 Colitis grade 1 Polyuria grade 1 |
| 15 | Toceranib | Enteritis grade 2 |
| 16 | CHOP | Neutropenia grade 2 Vomiting grade 3 Diarrhoea grade 3 Lethargy/fatigue grade 1 |
| 17 | Toceranib | Neutropenia grade 1 Diarrhoea grade 1 Colitis grade 1 Gastric ulceration grade 2 |
| 18 | CHOP | Vomiting grade 3 Colitis grade 1 Gastric ulceration grade 2 |
| 19 | Lomustine Toceranib Melphalan | Neutropenia grade 5 Haemoglobin grade 1 Diarrhoea grade 1 Colitis grade 2 |
| Cat | Carboplatin | Neutropenia grade 2 Haemoglobin grade 1 Thrombocytopenia grade 1 |

CHOP: cyclophosphamide, doxorubicin, vincristine, prednisone; CLOP: cyclophosphamide, lomustine, vincristine, prednisone; COP: cyclophosphamide, vincristine, prednisone; LOP: lomustine, vincristine, prednisone; UW 25: cyclophosphamide, doxorubicin, vincristine.
